# Papillary Tumor of the Pineal Region Presenting With Obstructive Hydrocephalus: A Diagnostic and Management Challenge

**DOI:** 10.7759/cureus.115863

**Published:** 2026-09-06

**Authors:** Lisset Urquiza Portilla, Annabell Castro, Lilian A Maturell Rojas, Yoniel Suarez-Guerrero, Nicolas Ahye, Bryan Lieber

**Affiliations:** 1 Department of Neurosurgery, Hospital Corporation of America (HCA) Florida Westside Hospital, HCA Healthcare, Plantation, USA; 2 Department of Dermatology, Universidad de Ciencias Médicas de La Habana, Havana, USA; 3 Department of Internal Medicine, Universidad de Ciencias Médicas de Guantánamo, Guantánamo, CUB; 4 Department of General Medicine, Asociación Española de Socorros Mutuos, Montevideo, URY; 5 Department of Neurosurgery, Hospital Corporation of America (HCA) Florida Westside/Northwest Hospitals, Plantation, USA

**Keywords:** cam5.2, case report, ck18, dna methylation profiling, endoscopic third ventriculostomy, immunohistochemistry, obstructive hydrocephalus, papillary tumor of the pineal region, pineal region neoplasm, ventriculoperitoneal shunt

## Abstract

We report a 45-year-old woman with a papillary tumor of the pineal region (PTPR), a neuroepithelial neoplasm of the pineal gland, who presented with a one-year history of headaches, visual disturbance, and gait impairment and was found to have a pineal mass with obstructive hydrocephalus. She underwent endoscopic third ventriculostomy (ETV) with biopsy; the initial radiologic impression favored pineocytoma. Immunohistochemistry was nondiagnostic on the limited sample, and the diagnosis of PTPR was established only after review at two academic neuropathology centers. DNA methylation profiling was obtained for definitive molecular classification, with the result not yet available for review at the time of reporting. The lesion was resected via an interhemispheric transcallosal approach, but the patient developed recurrent hydrocephalus due to ETV failure, requiring a ventriculoperitoneal shunt. She completed adjuvant radiotherapy and showed no recurrence on the most recent surveillance MRI. In this patient, PTPR posed two challenges: diagnostic overlap with pineocytoma that required specialized and molecular workup, and persistent hydrocephalus after ETV failure that ultimately required permanent cerebrospinal fluid diversion.

## Introduction

The pineal gland is a midline structure situated in the posterior third ventricle, bounded superiorly by the splenium of the corpus callosum and inferiorly by the quadrigeminal plate, with the cerebral aqueduct (aqueduct of Sylvius) immediately anteroinferior to it [1]. Because the aqueduct is the narrowest segment of the ventricular system and the only pathway for CSF to pass from the third to the fourth ventricle, even a small mass in this region can compress and occlude it, producing obstructive (non-communicating) hydrocephalus with proximal dilation of the lateral and third ventricles [1]. Because the cranium is a rigid, fixed-volume compartment containing brain tissue, blood, and CSF, any process that impairs CSF outflow raises intracranial pressure, consistent with the Monro-Kellie doctrine; clinically, this manifests as headache, nausea and vomiting, and visual disturbance and, if uncorrected, papilledema or an upward gaze palsy from tectal compression (Parinaud syndrome) [2].

Papillary tumor of the pineal region (PTPR) is a rare neuroepithelial neoplasm first described by Jouvet A et al. in 2003 and incorporated into the WHO classification of CNS tumors in 2007 [3,4]. It is thought to arise from the specialized ependymocytes of the subcommissural organ and is characterized by a papillary architecture with epithelial-like cells and consistent immunoreactivity for cytokeratins [5,6]. Fewer than 200 cases have been reported in the English literature, and the tumor occurs predominantly in adults in the third to fourth decades of life, although it can also affect children [6,7]. PTPR corresponds to CNS WHO grade 2 or 3; definitive histologic grading criteria remain undefined, and its biological behavior is notable for a high rate of local recurrence [6,8,9].

Diagnosing PTPR remains challenging because of substantial radiologic and histologic overlap with other pineal region tumors, particularly pineocytoma, papillary ependymoma, choroid plexus papilloma, and metastatic papillary carcinoma [5,10]. This overlap is compounded when tissue is obtained by endoscopic or stereotactic biopsy: a well-documented diagnostic trap arises when a small, limited sample is interpreted as pineocytoma, since the fragmented tissue may not represent the full architectural and immunophenotypic heterogeneity of the tumor [11]. Immunohistochemistry plays a critical role in differentiation: PTPR characteristically expresses cytokeratins (notably CK18 and CAM5.2), along with CD56, neuron-specific enolase (NSE), S100, and transthyretin, while typically remaining negative or only focally positive for glial fibrillary acidic protein (GFAP), synaptophysin, and chromogranin [3,5,11,12]. Given the variable immunophenotype, the 2021 WHO classification increasingly emphasizes molecular strategies, including DNA methylation profiling, for definitive CNS tumor classification [9].

Pineal region tumors frequently manifest with obstructive hydrocephalus secondary to aqueductal compression. Endoscopic third ventriculostomy (ETV) combined with biopsy represents a preferred initial approach, providing CSF diversion and tissue diagnosis while avoiding permanent shunting in most patients [13,14].

Here, we present a case of PTPR causing obstructive hydrocephalus through aqueductal compression that required multi-institutional neuropathologic review and molecular testing. An initially effective ETV failed following tumor resection, ultimately requiring secondary ventriculoperitoneal (VP) shunt placement.

## Case presentation

A 45-year-old woman presented with an approximately one-year history of progressively severe headaches, visual disturbance, and gait and balance impairment that had begun to limit her daily activities. Fundoscopic examination revealed papilledema with optic disc edema, and neurologic examination noted a mild upward gaze palsy, consistent with incomplete Parinaud syndrome; retraction-convergence nystagmus and light-near pupillary dissociation were not documented in the medical record. She had no known history of malignancy and an otherwise unremarkable medical history.

Non-contrast head CT demonstrated a pineal-region soft-tissue mass measuring approximately 15 × 12 mm, associated with third and lateral ventriculomegaly, mild transependymal CSF flow consistent with obstructive hydrocephalus, and mild cerebellar tonsillar crowding at the foramen magnum. Subsequent contrast-enhanced brain MRI confirmed a 13 × 12 × 13 mm lobulated, enhancing pineal lesion causing aqueductal obstruction (Figures 1-2); the initial radiologic differential diagnosis favored a pineal parenchymal tumor (pineocytoma).

**Figure 1:**
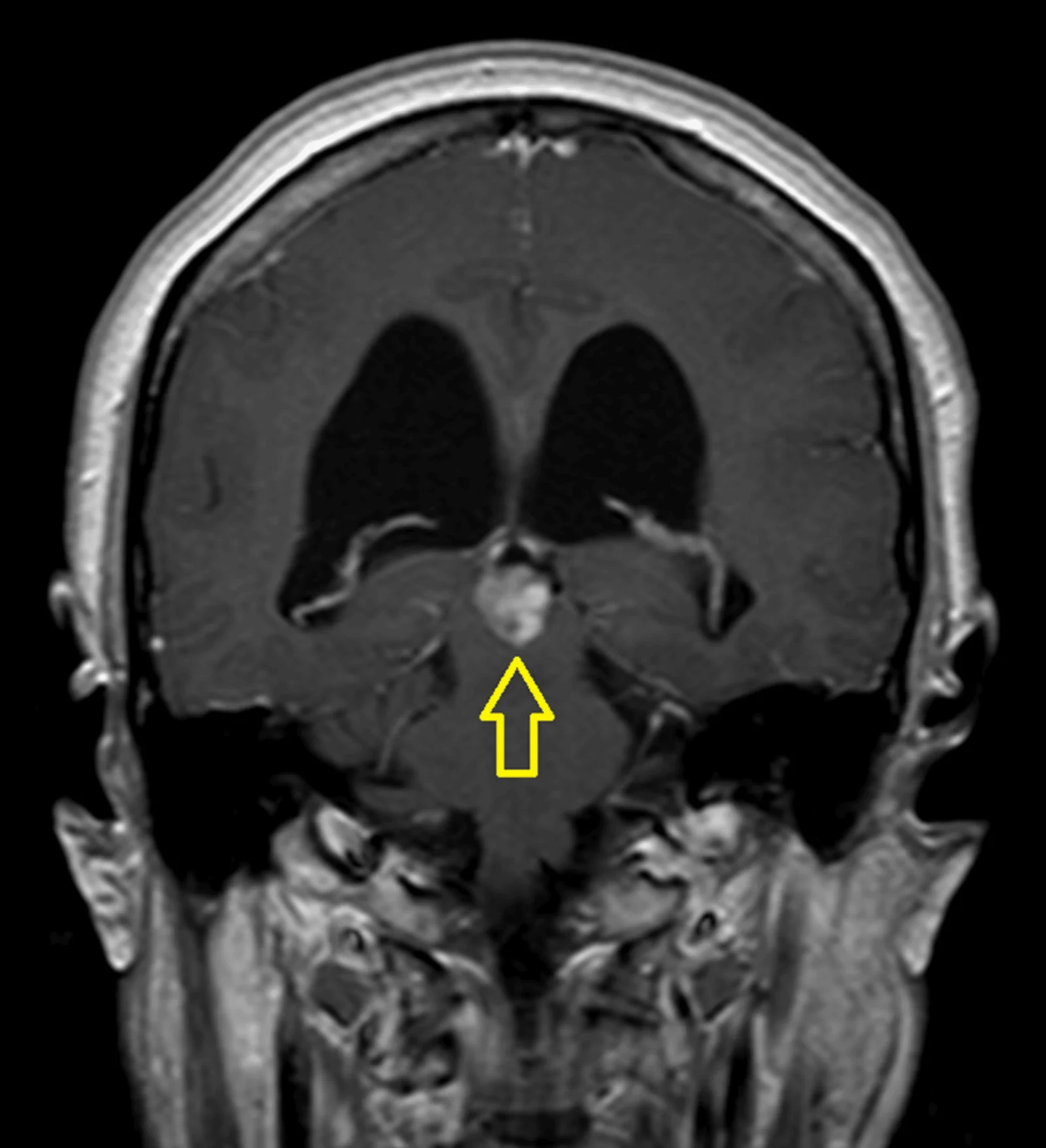
Preoperative coronal T1-weighted contrast-enhanced MRI of a pineal region mass. Contrast-enhanced coronal T1-weighted MRI revealing a heterogeneously enhancing mass in the pineal region (yellow arrow). The heterogeneous enhancement pattern and anatomic location raised initial concern for a pineal parenchymal tumor and prompted endoscopic third ventriculostomy with biopsy.

**Figure 2:**
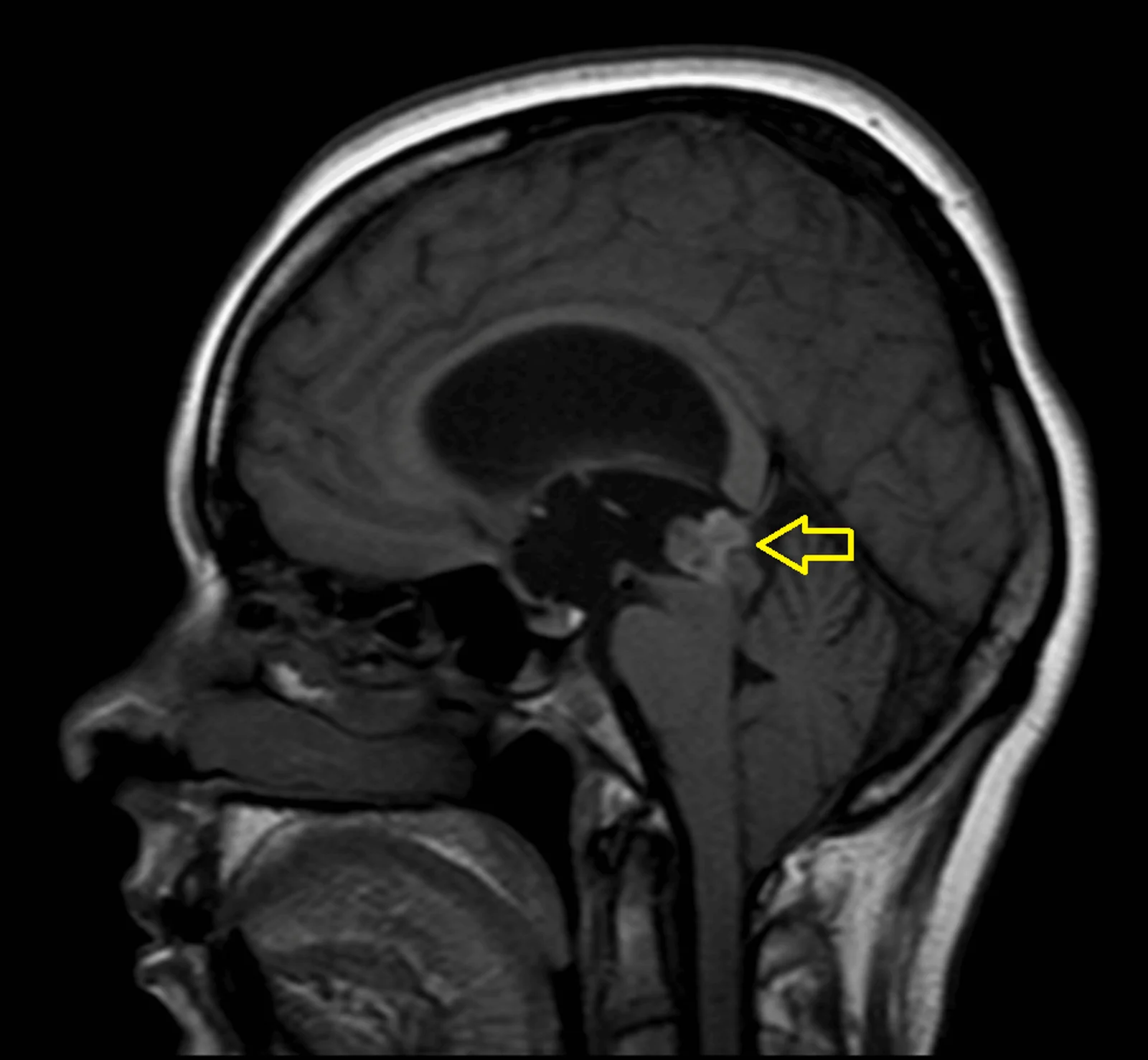
Preoperative sagittal T1-weighted MRI without contrast. Sagittal T1-weighted MRI without contrast showing a well-circumscribed, lobulated, slightly hyperintense mass in the pineal region (yellow arrow), exerting mass effect and effacing the cerebral aqueduct, with resultant dilation of the lateral and third ventricles.

Because of symptomatic obstructive hydrocephalus (Figure 3), the patient underwent ETV with endoscopic biopsy of the lesion.

**Figure 3:**
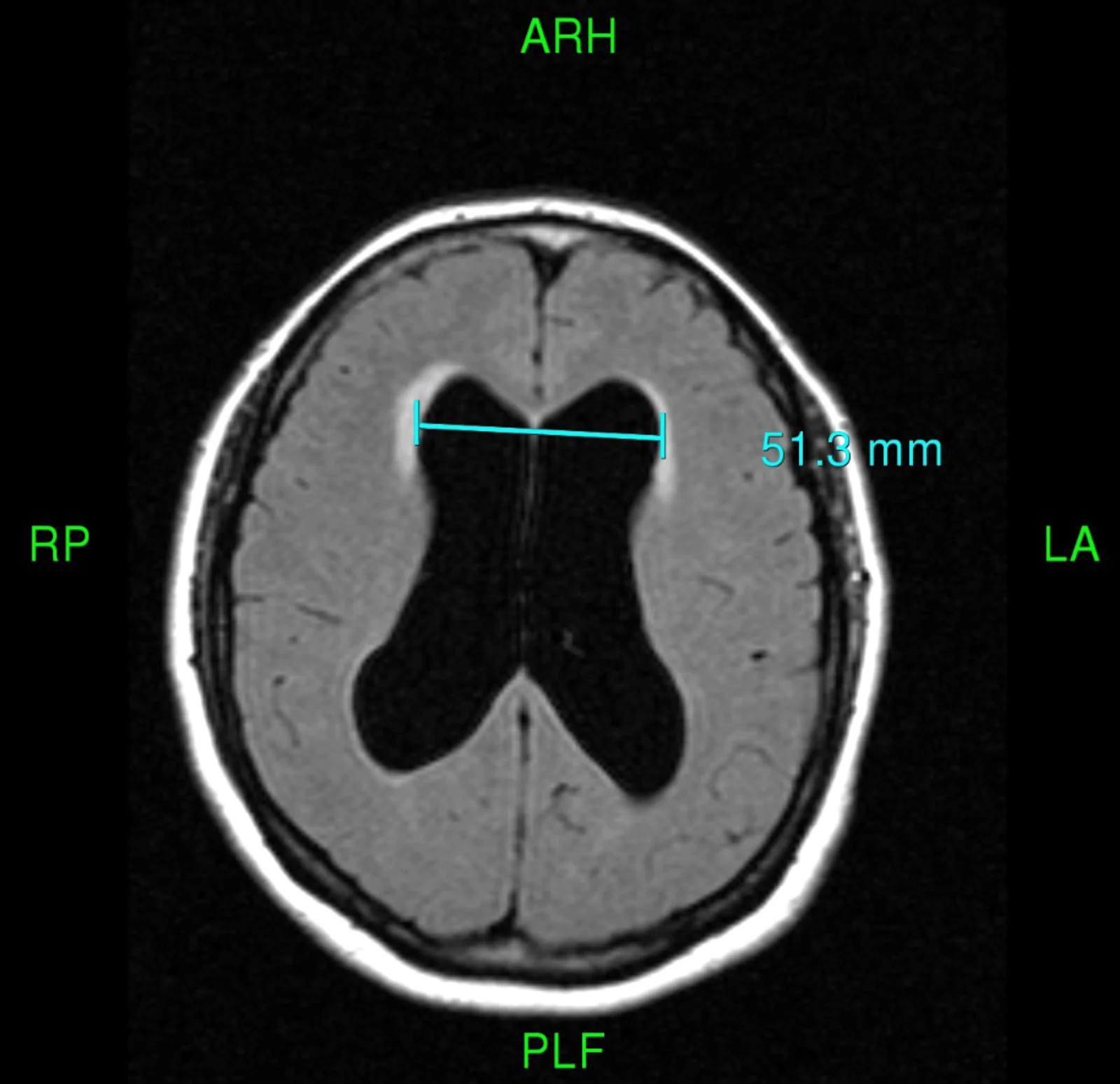
Preoperative axial MRI. Axial fluid-attenuated inversion recovery (FLAIR) image showing severe obstructive hydrocephalus with transependymal periventricular edema and a frontal horn diameter of 51.3 mm, findings indicating acute decompensation of chronic ventricular enlargement and supporting urgent cerebrospinal fluid diversion.

A postoperative CT scan demonstrated the expected right frontal burr-hole tract, interval reduction in ventricular size and transependymal edema, and a persistent pineal mass; however, persistent ventriculomegaly was still noted (Figure 4).

**Figure 4:**
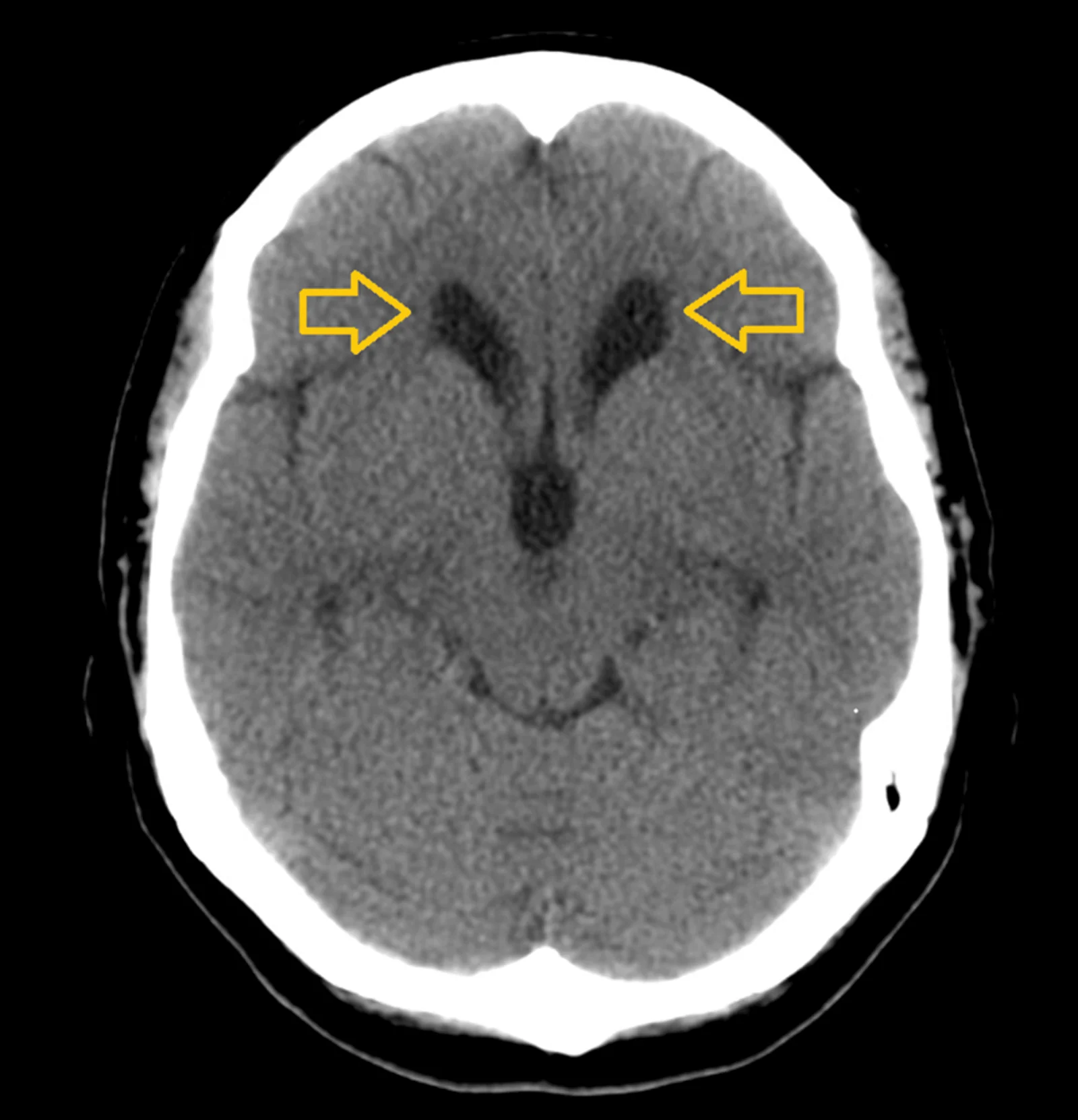
Postoperative non-contrast head CT. Axial CT image demonstrating persistent third ventriculomegaly and frontal horn dilation secondary to a persistent soft-tissue mass in the posterior third ventricle obstructing the cerebral aqueduct, necessitating subsequent CSF diversion.

The initial biopsy specimen was small. Immunohistochemistry showed tumor cells positive for CD56 and NSE, with focal weak synaptophysin positivity, and negative for GFAP and chromogranin, with a low Ki-67 proliferation index. As these findings were nondiagnostic for a specific entity, the tissue was sent for academic neuropathology consultation. The expert review described a papillary and epithelioid neoplasm (diffuse strong CK8/18; focal weak transthyretin; weak epithelial membrane antigen (EMA); strong diffuse CD56; weak NSE and synaptophysin; negative for neurofilament, NeuN, GFAP, and chromogranin) most consistent with PTPR, CNS WHO grade 2. The consultants noted that grading criteria for this rare tumor are not fully established and warned that the limited biopsy sample might not be representative of the entire lesion.

Definitive management was planned as surgical resection followed by radiotherapy. Definitive resection was deferred for approximately four months following pathologic confirmation of PTPR, reflecting the patient's preference to wait until her mother could travel from Mexico to be present for the surgery and to accommodate work-related scheduling, rather than any change in clinical urgency; she remained clinically improved after the ETV throughout this interval.

Preoperative surveillance MRI (approximately three months after the initial biopsy) measured the lesion at 1.7 cm in greatest dimension versus 1.3 cm initially; because the initial study was unavailable for direct comparison, true interval growth could not be reliably distinguished from measurement variability. Given the histological uncertainty at that point, the radiologic differential diagnosis was broadened to include PTPR, germinoma, teratoma, choriocarcinoma, pineal melanoma, and a proteinaceous pineal cyst.

Subsequently, she underwent craniotomy via an interhemispheric transcallosal approach for definitive resection of the pineal mass. Her early postoperative course was complicated by recurrent severe obstructive hydrocephalus attributed to failure of the previously performed ETV, requiring placement of a VP shunt approximately one week later. She subsequently developed low-pressure hydrocephalus symptoms, which improved following downward pressure adjustment of the programmable valve (Medtronic Strata II).

The resection specimen measured 1.8 × 1.5 × 0.5 cm, with histologic features (cellular neoplasm with round nuclei and amphophilic cytoplasm, discrete cell borders, growth in nodules and pseudopapillae, abundant hemorrhage, no necrosis, and rare mitoses at 1 per 10 high-power fields) and an immunohistochemical panel performed at a second academic center (Table 1) consistent with PTPR. Overall morphology and immunophenotype confirmed the diagnosis, and whole-genome DNA methylation profiling was recommended for definitive molecular subtyping.

**Table 1:** Comparative immunohistochemical panel across diagnostic stages. CAM5.2: Cytokeratin CAM5.2; CD56: Cluster of differentiation 56; CK8/CK18: Cytokeratins 8 and 18; EMA: Epithelial membrane antigen; GFAP: Glial fibrillary acidic protein; NeuN: Neuronal nuclear antigen; NSE: Neuron-specific enolase.

Marker	Initial biopsy (local pathology)	Academic consultation (same specimen)	Resection specimen (second academic center)
CD56	Positive	Strong, diffuse positive	Strong, diffuse positive
NSE	Positive	Weak positive	Positive
Synaptophysin	Focal weak positive	Weak positive	Negative
GFAP	Negative	Negative	Negative
Chromogranin	Negative	Negative	Negative
Ki-67 index	Low (not quantified)	Not reported	<2%
CK8/CK18	Not reported	Strong, diffuse positive	Positive
CAM5.2	Not reported	Not reported	Positive
Transthyretin	Not reported	Focal weak positive	Not reported
EMA	Not reported	Weak positive	Not reported
S100	Not reported	Not reported	Patchy positive
Neurofilament	Not reported	Negative	Negative in tumor cells
NeuN	Not reported	Negative	Not reported
Pan-cytokeratin	Not reported	Not reported	Negative

The patient completed adjuvant stereotactic radiotherapy. Serial postoperative surveillance MRIs demonstrated expected postoperative changes without residual or recurrent tumor and resolution of hydrocephalus, with the VP shunt effectively decompressing the ventricles. At the most recent surveillance MRI (Figures 5-6), approximately 15 months after the initial ETV and approximately nine and a half months after tumor resection, there was no evidence of tumor recurrence.

**Figure 5:**
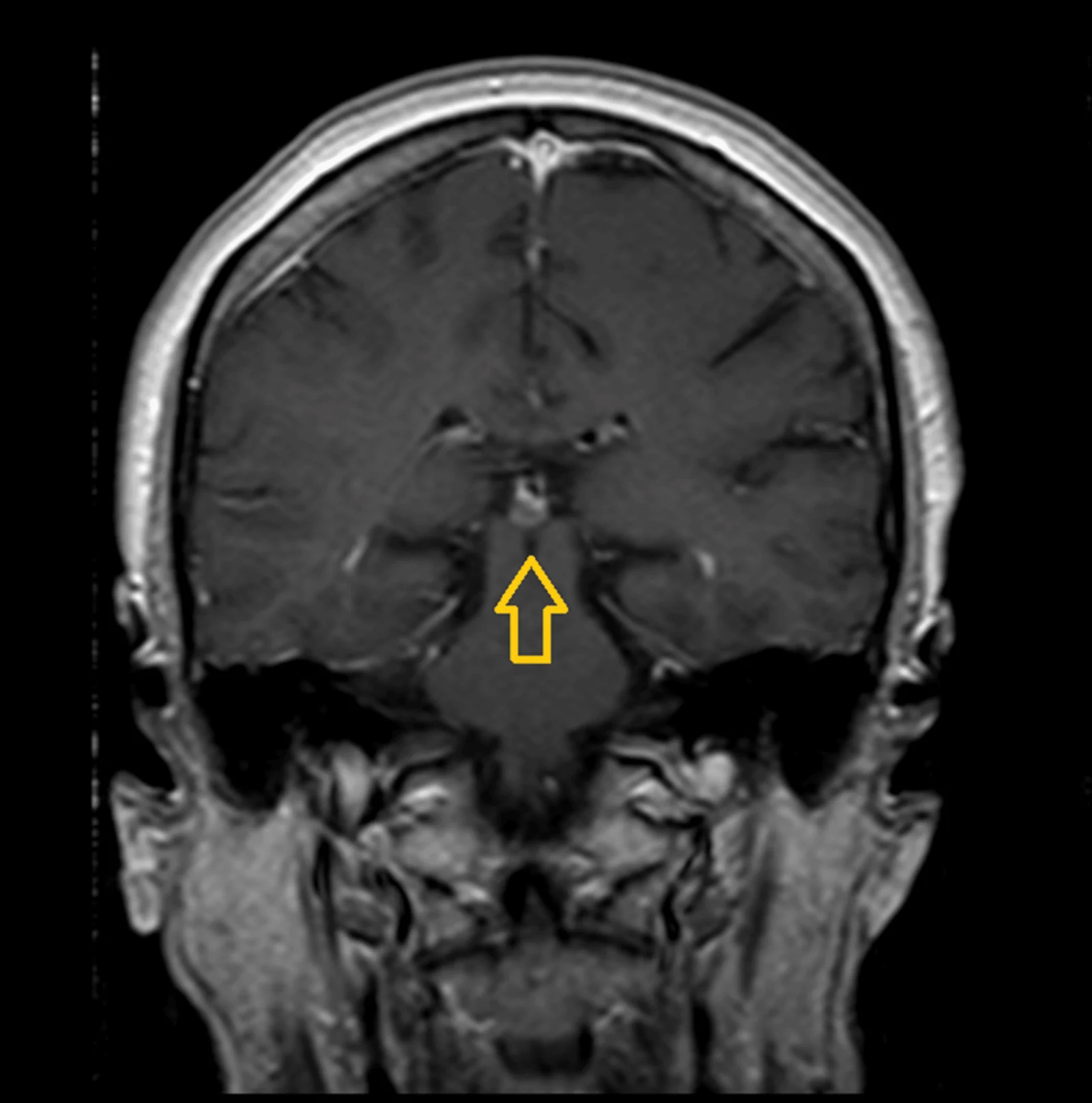
Postoperative surveillance brain MRI at 9 months. Approximately 15 months after the initial endoscopic third ventriculostomy and approximately nine and a half months after resection. Coronal contrast-enhanced T1-weighted MRI demonstrating expected postoperative changes following resection of the pineal region tumor. No evidence of residual or recurrent pathologic enhancement is observed, and the lateral ventricles show significant decompression compared with preoperative imaging.

**Figure 6:**
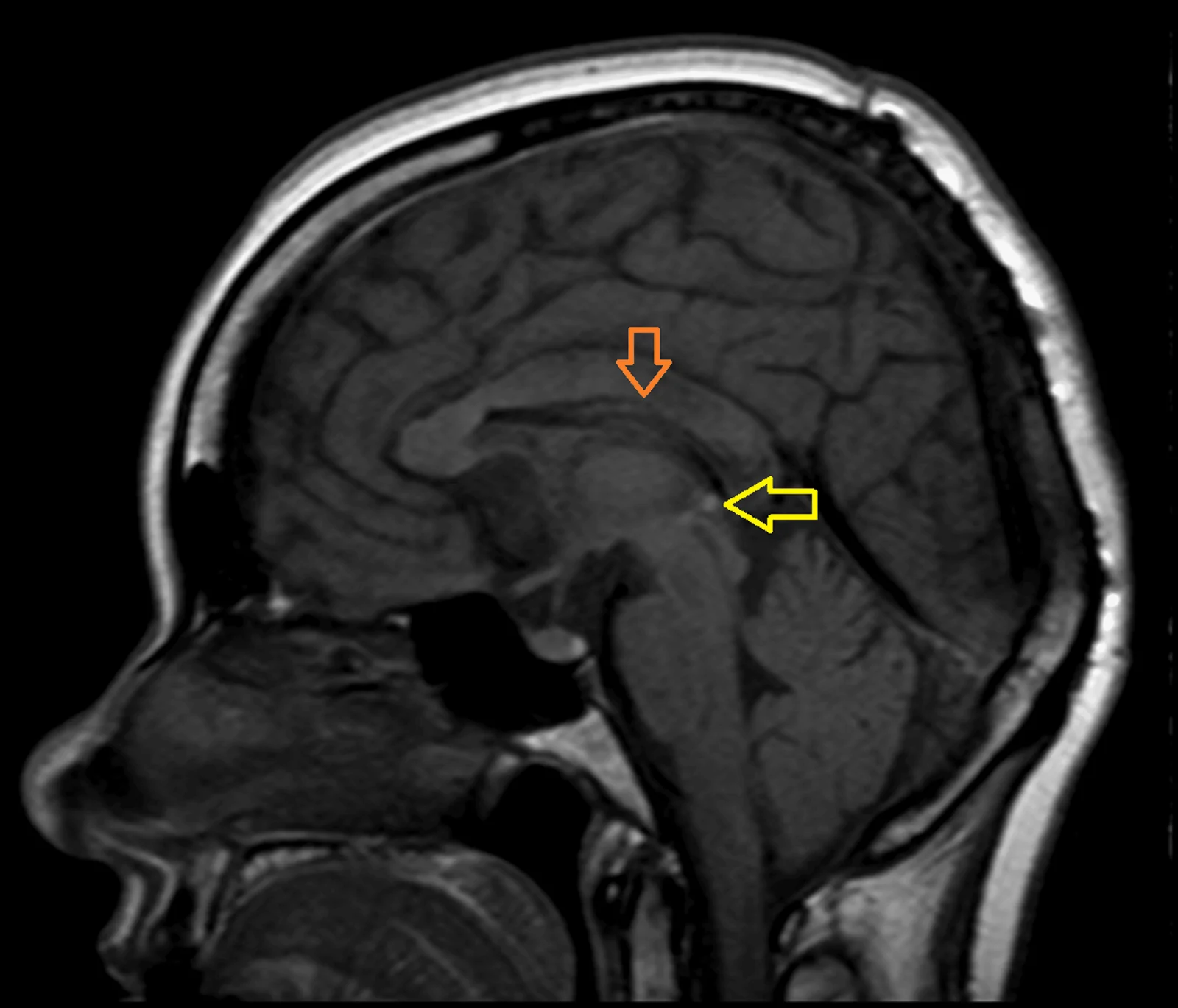
Postoperative surveillance sagittal brain MRI at 9 months. Sagittal T1-weighted MRI without contrast at nine and a half months after resection, showing interval decompression of the third ventricle (orange arrow) compared with preoperative imaging and no residual or recurrent mass in the pineal region (yellow arrow).

Clinically, her presenting symptoms had fully resolved, her visual disturbances had improved, and she returned to full functional status (Table 2).

**Table 2:** Timeline of key diagnostic and therapeutic milestones. ETV: Endoscopic third ventriculostomy; FLAIR: Fluid-attenuated inversion recovery; PTPR: Papillary tumor of the pineal region; VP: Ventriculoperitoneal.

Time point	Event	Key finding
~August 2023	Symptom onset	Progressive headache, visual disturbance, and gait and balance impairment
July 24, 2024	Non-contrast head CT	15 × 12 mm pineal mass; obstructive hydrocephalus
August 2, 2024	Brain MRI with and without contrast	13 × 12 × 13 mm pineal lesion; initial impression favored pineocytoma
August 29, 2024 (reference, month 0)	ETV with endoscopic biopsy	Small initial specimen; nondiagnostic on local pathology
September 12, 2024 (~2 weeks)	Postoperative head CT	Decreased ventriculomegaly; persistent pineal mass
October 4, 2024 (~1.2 months)	Final biopsy pathology (academic consultation)	PTPR, CNS WHO grade 2
December 4, 2024 (~3.2 months)	Preoperative surveillance MRI	Lesion measured 1.7 × 1.4 × 1.2 cm versus 1.3 × 1.2 × 1.3 cm initially; measurement variability, not confirmed growth
February 6, 2025 (~5.3 months)	Interhemispheric transcallosal resection	Gross total resection
February 11, 2025 (~5 days post-resection)	VP shunt placement	Recurrent hydrocephalus secondary to ETV failure
April 2025 to June 2025	Adjuvant stereotactic radiotherapy	Completed approximately 2 to 4 months after resection
November 25, 2025 (~14.9 months post-ETV; ~9.6 months post-resection)	Surveillance MRI	No residual or recurrent tumor in the pineal bed; stable nonspecific FLAIR signal in the corpus callosum

## Discussion

PTPR presents two major clinical hurdles: diagnostic ambiguity requiring specialized pathology or molecular confirmation, and persistent obstructive hydrocephalus that may outlast initial endoscopic management.

Clinically, the presenting symptoms of headache, visual disturbance, and gait imbalance reflect the two mechanisms outlined in the Introduction: aqueductal compression by the pineal mass and the resulting rise in intracranial pressure. Recognizing this symptom complex as a possible marker of obstructive hydrocephalus, rather than as a nonspecific complaint, is what prompted the neuroimaging that led to the diagnosis in this patient.

Radiologically, the initial differential diagnosis frequently favors pineocytoma, a well-documented diagnostic trap due to overlapping imaging features and histopathology on small tissue samples [11]. The cytokeratin-positive immunophenotype in this case, together with GFAP negativity, distinguishes PTPR from its principal mimics, notably arguing against papillary ependymoma [5,11]. This diagnostic trajectory, ultimately requiring multi-institutional review and molecular profiling for definitive classification, reflects the integrated, molecularly guided approach advocated by the 2021 WHO classification of CNS tumors [9].

PTPR corresponds to WHO grade 2 or 3, but grading criteria remain undefined, and the entity is characterized by frequent local recurrence, reported to exceed 70% at six years, which underlies the common use of adjuvant radiotherapy [6,8]. However, the largest multicenter series to date (44 patients) found that only gross total resection and younger age at diagnosis were associated with longer overall survival, while neither adjuvant radiotherapy nor chemotherapy had a statistically significant effect on overall or progression-free survival; median progression-free survival in that series was 58.1 months, and median overall survival was not reached [15]. Chemotherapy currently has no established role in the primary treatment of PTPR and has been reserved, with variable and largely anecdotal results, for recurrent or refractory disease.

Our patient completed adjuvant stereotactic radiotherapy and showed no evidence of recurrence on the most recent surveillance MRI, approximately 15 months after the initial ETV and approximately nine and a half months after resection. Given the recurrence risk, continued long-term imaging surveillance remains essential, and the current disease-free interval should be interpreted cautiously.

From a management standpoint, ETV combined with endoscopic biopsy is a preferred strategy for pineal tumors with obstructive hydrocephalus, achieving high rates of CSF diversion and diagnosis while sparing most patients a permanent shunt; reported subsequent shunt rates are low (approximately 8%) [13,14,16]. This patient, however, developed recurrent severe hydrocephalus after tumor resection owing to failure of the previously performed ETV and required a VP shunt, with subsequent low-pressure hydrocephalus that resolved only after downward adjustment of the programmable valve. The course is a reminder that a functioning ETV can fail later in the disease course, particularly around resection, and that clinicians should anticipate the possible need for shunting and careful valve titration.

Because PTPR can rarely disseminate along the neuraxis, current imaging guidance recommends contrast-enhanced evaluation of the entire craniospinal axis and CSF cytology, where feasible, at the time of diagnosis to exclude leptomeningeal or spinal seeding [17]. This screening was not performed in the present case.

A comparative overview of pineal region tumors and their principal distinguishing features, useful in broadening the differential diagnosis, is provided in Table 3 [18].

**Table 3:** Comparative overview of pineal region tumors. CAM5.2: Antibody clone against low-molecular-weight cytokeratins; CD56: Cluster of differentiation 56; CD117: Cluster of differentiation 117 (c-KIT); CK8/18: Cytokeratins 8 and 18; EMA: Epithelial membrane antigen; GFAP: Glial fibrillary acidic protein; NSE: Neuron-specific enolase; OCT4: Octamer-binding transcription factor 4; PPTID: Pineal parenchymal tumor of intermediate differentiation; PTPR: Papillary tumor of the pineal region; PLAP: Placental alkaline phosphatase.

Tumor	WHO grade	Typical age/demographics	Key histopathologic/immunohistochemical features	Treatment	Prognosis
PTPR	2 or 3	Adults, mean age approximately 30s; rare in children	Papillary architecture; positive for CK8/18, CAM5.2, CD56, NSE; negative for GFAP and chromogranin	Gross total resection; radiotherapy commonly used, though not shown to significantly improve survival	High recurrence (up to approximately 70% at 5 to 6 years); 5-year overall survival approximately 73%
Pineocytoma	1	Adults	Well-differentiated, pineocytomatous rosettes; positive for synaptophysin; negative for GFAP	Gross total resection, usually without adjuvant therapy	Excellent; 5-year survival approximately 100%, 20-year survival approximately 76%
PPTID	2 or 3	Adults, wide age range	Histology intermediate between pineocytoma and pineoblastoma	Gross total resection plus radiotherapy	Intermediate and variable, dependent on grade and treatment response
Pineoblastoma	4	Predominantly children	Primitive small round blue cell tumor, high mitotic activity, prone to CSF dissemination	Maximal safe resection, craniospinal radiotherapy, multiagent chemotherapy	Poor; 5-year survival under 60%, worse in young children
Papillary ependymoma	2 (ependymoma grading)	Any age	Perivascular pseudorosettes, dot-like paranuclear EMA positivity; positive for GFAP (key feature distinguishing it from PTPR)	Gross total resection; radiotherapy for subtotal resection or higher grade	Variable, generally more favorable than pineoblastoma
Choroid plexus papilloma	1	More often children when supratentorial	Papillary fronds lined by a single layer of cuboidal epithelium resembling normal choroid plexus; positive for cytokeratin and transthyretin	Gross total resection, usually curative	Excellent for grade 1; choroid plexus carcinoma (grade 3) has a substantially worse prognosis
Germinoma	Not graded on the WHO CNS scale	Over 90% of patients younger than 20 years; male predominance in the pineal region	Large primitive germ cells with a lymphocytic infiltrate; positive for PLAP, OCT4, and CD117	Highly radiosensitive and chemosensitive; radiotherapy with or without chemotherapy; surgery mainly for biopsy	Excellent; 5-year survival at least 90%

Limitations include those inherent to a single case, the relatively short post-resection follow-up, the absence of craniospinal MRI or CSF cytology to formally exclude occult spinal or leptomeningeal dissemination, and the fact that the result of DNA methylation profiling, obtained but not yet available for review at the time of reporting, remains pending for definitive molecular classification.

## Conclusions

PTPR should be considered in the differential diagnosis of pineal masses, particularly when initial imaging or biopsy suggests pineocytoma. Cytokeratin immunohistochemistry remains a mainstay of diagnosis, while DNA methylation profiling, obtained in this patient but with the result not yet available for review at the time of reporting, is increasingly important for definitive molecular classification. Furthermore, because ETV failure can occur following tumor resection, clinicians should remain vigilant for recurrent hydrocephalus requiring secondary CSF diversion. Finally, the high rate of local recurrence in PTPR underscores the necessity of long-term radiologic surveillance despite successful resection and adjuvant radiotherapy.
